# Confocal Laser Endomicroscopy and Optical Coherence Tomography for the Diagnosis of Prostate Cancer: A Needle-Based, In Vivo Feasibility Study Protocol (IDEAL Phase 2A)

**DOI:** 10.2196/resprot.9813

**Published:** 2018-05-21

**Authors:** Abel Swaan, Christophe K Mannaerts, Matthijs JV Scheltema, Jakko A Nieuwenhuijzen, C Dilara Savci-Heijink, Jean JMCH de la Rosette, R Jeroen A van Moorselaar, Ton G van Leeuwen, Theo M de Reijke, Daniel Martijn de Bruin

**Affiliations:** ^1^ Department of Urology Academic Medical Center University of Amsterdam Amsterdam Netherlands; ^2^ Department of Biomedical Engineering and Physics Academic Medical Center University of Amsterdam Amsterdam Netherlands; ^3^ Department of Urology VU University Medical Center VU University Amsterdam Netherlands; ^4^ Department of Pathology Academic Medical Center University of Amsterdam Amsterdam Netherlands; ^5^ Academic Medical Center University of Amsterdam Amsterdam Netherlands; ^6^ Department of Urology Istanbul Medipol University Istanbul Turkey

**Keywords:** confocal laser endomicroscopy, optical coherence tomography, prostate, prostatic neoplasms biopsy, prostatectomy, microscopy, histology, optical imaging

## Abstract

**Background:**

Focal therapy for prostate cancer has been proposed as an alternative treatment to whole-gland therapies in selected men to diminish side effects in localized prostate cancer. As nowadays imaging cannot offer complete prostate cancer disease characterization, multicore systematic biopsies are recommended (transrectal or transperineal). Optical imaging techniques such as confocal laser endomicroscopy and optical coherence tomography allow in vivo, high-resolution imaging. Moreover, they can provide real-time visualization and analysis of tissue and have the potential to offer additive diagnostic information.

**Objective:**

This study has 2 separate primary objectives. The first is to assess the technical feasibility and safety of in vivo focal imaging with confocal laser endomicroscopy and optical coherence tomography. The second is to identify and define characteristics of prostate cancer and normal prostate tissue in confocal laser endomicroscopy and optical coherence tomography imaging by comparing these images with the corresponding histopathology.

**Methods:**

In this prospective, in vivo feasibility study, needle-based confocal laser endomicroscopy and optical coherence tomography imaging will be performed before transperineal template mapping biopsy or radical prostatectomy. First, confocal laser endomicroscopy and optical coherence tomography will be performed in 4 patients (2 for each imaging modality) undergoing transperineal template mapping biopsy to assess the feasibility and safety of confocal laser endomicroscopy and optical coherence tomography. If proven to be safe and feasible, confocal laser endomicroscopy and optical coherence tomography will be performed in 10 patients (5 for each imaging modality) undergoing radical prostatectomy. Confocal laser endomicroscopy and optical coherence tomography images will be analyzed by independent, blinded observers. Confocal laser endomicroscopy– and optical coherence tomography–based qualitative and quantitative characteristics and histopathology will be compared. The study complies with the IDEAL (Idea, Development, Exploration, Assessment, Long-term study) stage 2a recommendations.

**Results:**

At present, the study is enrolling patients and results and outcomes are expected in 2019.

**Conclusions:**

Confocal laser endomicroscopy and optical coherence tomography are promising optical imaging techniques that can visualize and analyze tissue structure, possible tumor grade, and architecture in real time. They can potentially provide real-time, high-resolution microscopic imaging and tissue characteristics of prostate cancer in conjunction with magnetic resonance imaging or transrectal ultrasound fusion-guided biopsy procedures. This study will provide insight into the feasibility and tissue-specific characteristics of confocal laser endomicroscopy and optical coherence tomography for real-time optical analysis of prostate cancer.

**Trial Registration:**

ClinicalTrials.gov NCT03253458; https://clinicaltrials.gov/ct2/show/NCT03253458 (Archived by WebCite at http://www.webcitation.org/6z9owM66B)

**Registered Report Identifier:**

RR1-10.2196/9813

## Introduction

Prostate cancer (PCa) is the leading noncutaneous cancer in men and the third cause of cancer-related death [1]. To date, patients with a clinical suspicion of PCa, based on elevated serum prostate-specific antigen (PSA) and/or suspicious digital rectal examination (DRE), are recommended to undergo transrectal ultrasound (TRUS; +/− multiparametric magnetic resonance imaging, mpMRI)–guided systematic biopsies [2]. This work-up for PCa diagnosis carries some important drawbacks. Due to the heterogeneous nature of PCa, this procedure has a known risk of missing PCa lesions or underestimating PCa aggressiveness, besides overdiagnosis of insignificant lesions [3,4]. In the last decade, the diagnostic pathway for PCa has, therefore, moved more and more into imaging-based targeted biopsies instead of random systematic biopsies. Reliable prostate imaging is key for the reduction of unnecessary biopsies, insignificant PCa detection, increasing detection of significant PCa, reducing the number of cores, and to facilitate monitoring during active surveillance. Moreover, reliable imaging would play a pivotal role in treatment planning, and monitoring of focal treatment for low- to intermediate-risk localized PCa [5-8]. Especially, mpMRI of the prostate has evolved as an increasingly appealing tool in the PCa diagnostic armamentarium and is recommended in men with suspicion of PCa following a negative initial biopsy, and currently, it is even proposed to select patients for biopsies [2,5]. For focal therapy, in which the aim is to target treatment of significant disease with minimal toxicity, accurate disease identification, localization, demarcation, and grading of a lesion are essential. Focal therapy selection with mpMRI-targeted biopsies may be an option in experienced hands, but to date, there is a substantial proportion of false positives in lesions 3/5 or 4/5 scored with the prostate imaging reporting data system (PI-RADS) [9]. Moreover, the assessment of mpMRI-negative areas or the prostate as a whole using a transperineal prostate mapping biopsy using a template-guided approach is recommended [2,10,11]. Transperineal template mapping biopsies (TTMB) are able to sample the prostate at every 5 mm, and coordinates are correlated to the tumor location. Limitations of this procedure are the large numbers of cores needed per prostate, the rate of urinary retentions, and the operating room time with its accompanying hospital admission [12,13]. Moreover, pathologists face a substantial increase in workload with a high number of biopsies, which often turn out to be benign.

Optical imaging technologies offer real-time imaging with excellent spatial and temporal resolution and are easily integrated into the operating room. In conjunction with mpMRI/TRUS-fusion image targeted biopsy, these real-time technologies in a needle-based form could provide valuable information for tissue characteristics. Adding real-time, in vivo diagnostic information of prostate tissue structure and architecture to already known information could improve PCa disease characterization. Optical imaging has the potential to make the diagnostic procedure less invasive, speed up the pathway, and reduce the currently existing workload of histopathological analysis.

**Figure 1:**
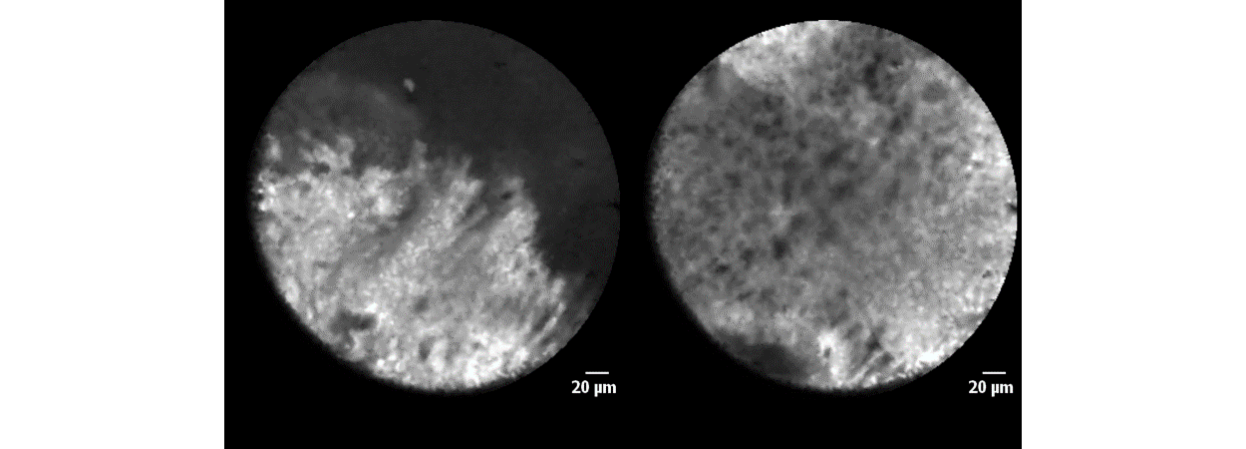
Two examples of confocal laser endomicroscopy (CLE) images with Cellvizio AQ-flex 19 probe of ex vivo prostate tissue soaked in fluorescein solution for 2 min.

**Figure 2:**
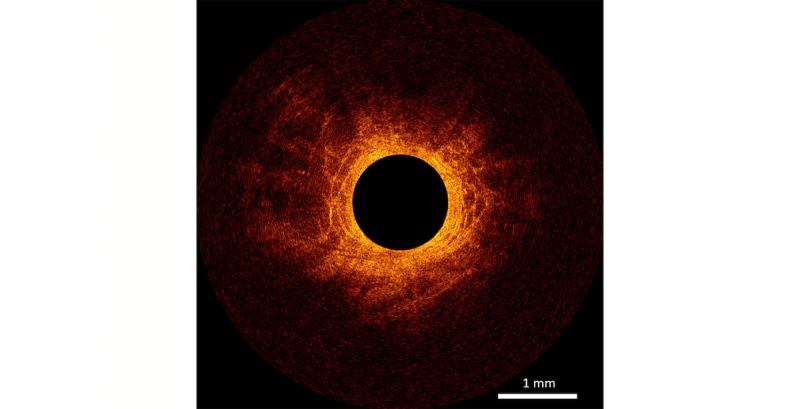
One b-scan of fixated ex vivo prostate tissue visualized with optical coherence tomography (OCT) with C7-XRtm Imaging System interfaced to a C7 Dragonflytm Imaging Probe (St. Jude Medical, St. Paul, Minnesota, USA).

Two optical imaging techniques currently used for needle-based optical biopsies are confocal laser endomicroscopy (CLE) and optical coherence tomography (OCT) [14-17]. CLE and OCT differ in background technology and image geometry and, therefore, show different images of the scanned tissue, see Figures 1 and 2.

CLE uses low-power laser bundles in a fiber optic probe, which can be inserted into the lumen of a needle to obtain real-time microscopic images of the tissue that is investigated. Backscattered light, from one specific tissue plane, is focused through a pinhole, whereas the backscattered light from surrounding tissue is rejected. This leads to high-resolution imaging of one specific plane of tissue in focus. The fluorescent light originates from the fluorescent dye nested in the extracellular matrix after topical or intravenous application. The most commonly used fluorescent dye is fluorescein. CLE is under investigation for gastrointestinal, urothelial, and pulmonary diseases, whereas for PCa, so far, only one study on CLE has been reported [14,18-20]. Lopez et al performed CLE during robot-assisted laparoscopic prostatectomy (RALP) in 21 patients to investigate the ability of CLE to assess surgical margins and nerve tissue with promising CLE-based characteristics of prostatic and periprostatic tissue [20]. In addition, no adverse events were reported related to the CLE procedure. However, these authors did not assess the ability to differentiate malignant from benign prostate cells.

OCT is the optical equivalent of ultrasound imaging, based on the backscattering of near-infrared light. Flexible OCT probes, which can be inserted into a needle lumen, enable side looking real-time imaging with an axial resolution up to 10 μm and an effective penetration depth of around 2 mm [21]. Cross-sectional images are generated using an automated pullback system while the probe is rotating a small laser light bundle over the tissue. Within urology, OCT has been applied in evaluating malignancy of bladder, upper urinary tract, kidney, testes, and prostate lesions [22]. In PCa, OCT has been applied for intraoperative identification of neurovascular bundles, surgical margins, and extracapsular extension with the goal to preserve patient’s functional and oncological outcomes [23-26]. A limited number of studies have looked at OCT’s diagnostic role in differentiating benign and malignant microscopic tissue of the prostate gland. Muller et al demonstrated with the use of a histopathologic validation tool that ex vivo needle-based OCT measurements of radical prostatectomy specimens could differentiate between cancer and healthy prostate tissue [27-29]. The quantitative analysis of the OCT signal by means of the attenuation coefficient was significantly higher in malignant compared with benign tissue with an area under the curve ranging from 0.64 to 0.89 depending on the histopathological analysis used [29].

The development of CLE and OCT toward real-time optical biopsies of prostate carcinoma may lead to advances in diagnosis and (focal therapy) treatment. Following phase 2a of the IDEAL criteria [30], we have separated the study protocol into 2 sequential aims with different procedures.

Procedure 1 aims to evaluate the technical feasibility of needle-based in vivo imaging with CLE and OCT in the prostate.

Procedure 2 aims to describe characteristics to be used for PCa detection, which allows us to create an atlas of CLE and OCT characteristics of normal and malignant prostate tissue based on a one-to-one comparison with histology.

## Methods

### Study Objectives

The objective of procedure 1 is to assess the technical feasibility and safety of in vivo, needle-based, focal imaging of prostate tissue with CLE and OCT.

Procedure 2 has as primary objective to identify and define characteristics of PCa on CLE and OCT images. The secondary objectives are to correlate CLE and OCT images with histopathology, to develop an in vivo CLE and OCT image atlas of the prostate, and to assess procedure-related adverse events. The atlas will differentiate prostate tissues including benign glands, cystic atrophy, regular atrophy, stroma, inflammation, fat as well as different grades of malignant tissue using the Gleason score. The procedure-related adverse events will be evaluated using the Common Terminology Criteria for Adverse Events.

### Study Design

This study is an investigator-initiated, multicenter, prospective in vivo feasibility study, using in vivo needle-based imaging methods with CLE and OCT. Approval of the local institutional review board (IRB) has been obtained for the study protocol under registry number: NL57326.018.17 on July 7, 2017, and the study was registered on the clinicaltrials.gov database (NCT03253458) on August 18, 2017. Any amendments to the trial protocol will be submitted for review by the IRB. Trial registrations will be updated, and participants will be informed about the risks and benefits of participation both verbally by one of the investigators and in writing in the form of an extensive patient information brochure. Participants will only be included after written informed consent has been obtained. Patients can leave the study at any time for any reason if they wish to do so without any consequences. The investigator can decide to withdraw a subject from the study for urgent (medical) reasons. Patient data will be anonymized and stored in a secure database.

**Figure 3:**
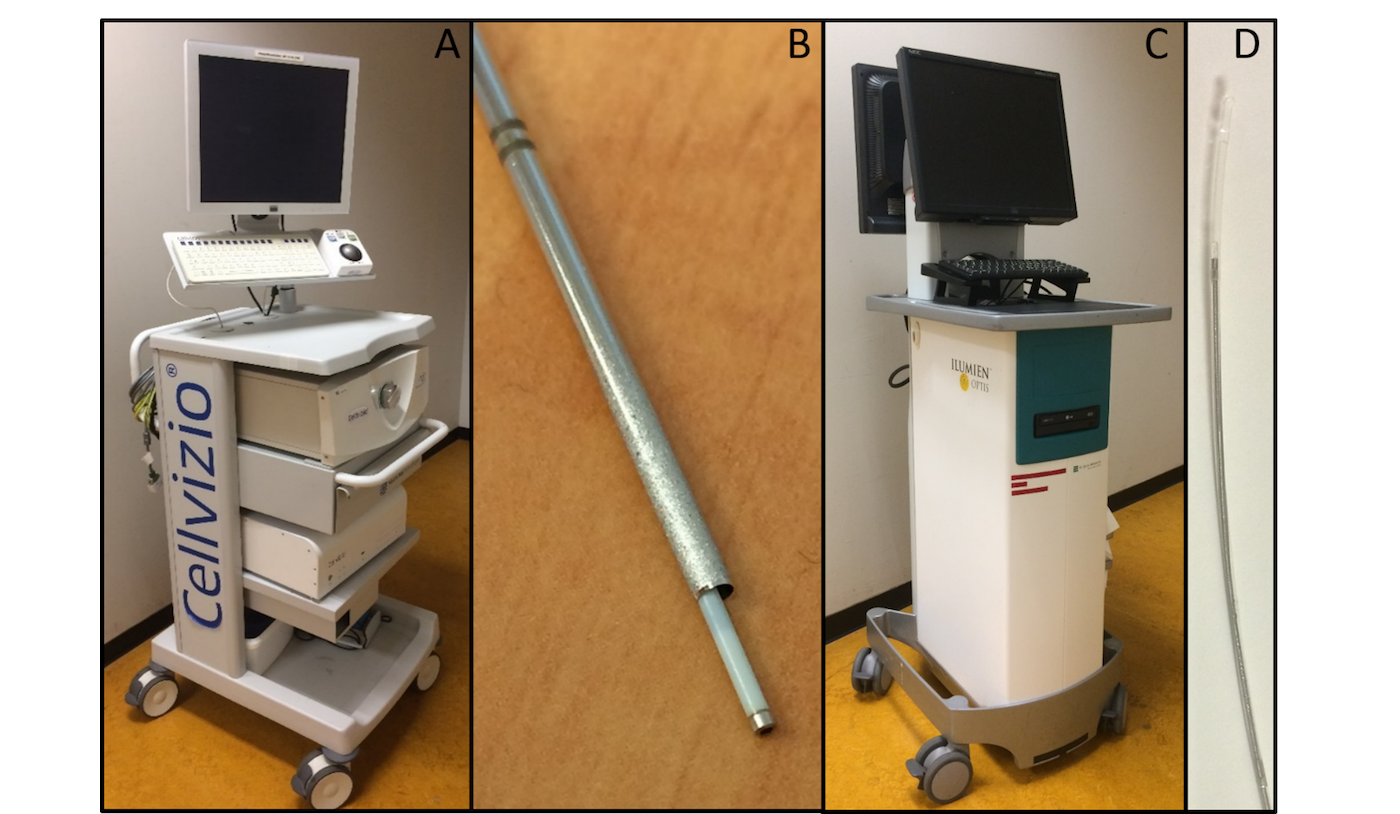
A: Cellvizio CLE system, B: Cellvizio confocal Miniprobe (AG-Flex 19) through the 17-gauge introducer needle, C: St. Jude Lightlab optical coherence tomography (OCT) system, D: St. Jude OCT probe with a 0.9 mm diameter.

The study design consists of 2 sequential procedures. CLE images are recorded with the AG-Flex 19 fiber optic mini probe-based system (Cellvizio System, Mauna Kea Technologies, Paris, France) with an outer diameter of 0.9 mm, a field of view of 325 µm, and a resolution of 3.5 µm. OCT images are recorded with a small rotating C7 Dragonfly Imaging Probe using the Light Lab OCT system (St. Jude Medical, Saint Paul, Minnesota, USA). Both devices and probes are illustrated in Figure 3.

For CLE imaging, a fluorescent contrast agent is needed to stain the extracellular matrix. Fluorescein (fluorescein sodium, Fresenius Kabi, Zeist, the Netherlands), a nontoxic and commonly used fluorescent dye, will be administered intravenously through an intravenous cannula [31]. Two times a bolus of 2.5 mL of 10% sodium fluorescein will be administered, one bolus per CLE measurement. The probes are transperineally introduced through a 17-gauge needle under ultrasound guidance. CLE images are recorded at a scan rate of 12 frames per second during a push and scan technique after placing the probe in direct contact with prostate tissue.

The OCT probe will be placed with a trocar needle in the prostate tissue under ultrasound guidance. After removal of the trocar needle, the inner part of the probe, the laser lens system is automatically pulled back while it is rotating, which creates a 3D image of the tissue.

In procedure 1, patients scheduled for TTMB will undergo in vivo CLE or OCT imaging, before colocalized biopsy for standard histopathological assessment.

If it is shown in procedure 1 that in vivo CLE and OCT imaging are technically feasible and safe to perform, then procedure 2 will be initiated. In procedure 2, patients scheduled for RALP will undergo in vivo CLE or OCT imaging during surgery, before prostate removal. In general, 2 recordings of 90 s each will be made for the per-patient chosen modality. Recorded CLE and OCT imaging will be analyzed, at a later stage, by blinded independent observers and compared with the corresponding histopathologic evaluation of the prostatectomy specimen. Histopathological analysis is performed according to the standard clinical protocol and will be performed by a uropathologist, blinded for OCT and CLE imaging results. The uropathologist will, next to the standard examination procedure, perform a detailed reporting method; prostate tissue will be analyzed and annotated for various structures (benign glands, cystoid atrophy, regular atrophy, stroma, malignant tissue using the Gleason score, inflammation, and fat) on the whole mount histology slice or biopsies specimens. Histopathology is correlated with CLE and OCT data in a 3D computer environment. Adverse events are registered with a follow-up of 30 days.

### Population

**Figure 4:**
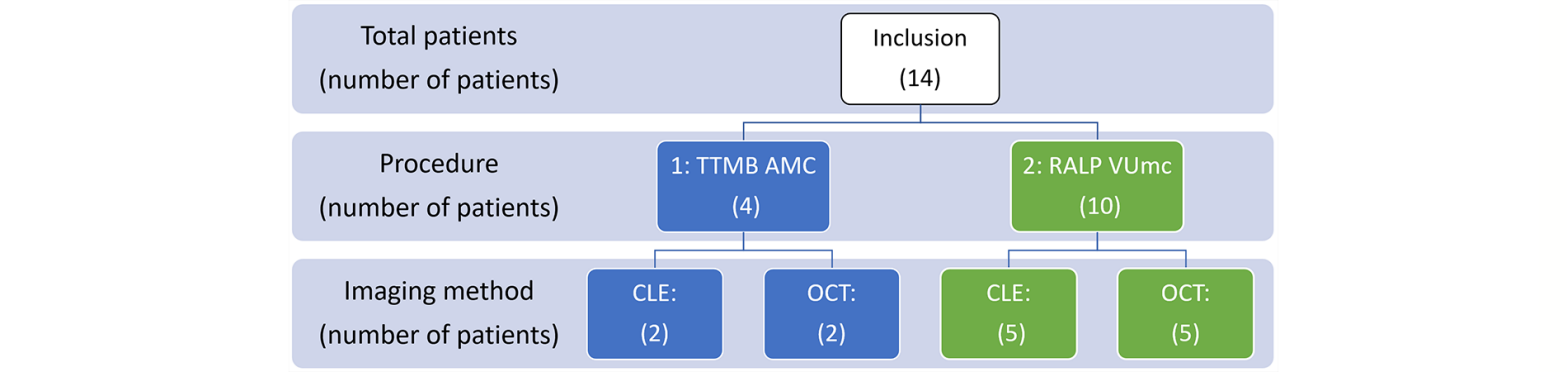
Study design, procedure 2: robot-assisted laparoscopic prostatectomy (RALP) will only start after a positive outcome of procedure 1: transperineal template mapping biopsy (TTMB). CLE: confocal laser endomicroscopy; OCT: optical coherence tomography.

Patients (aged ≥18 years) who are indicated for a TTMB will be included for study procedure 1. All patients will be recruited in the AMC Hospital (Amsterdam, the Netherlands), and all study procedures will be performed in this institution. A total of 14 patients will be included in this study (Figure 4). Four patients will be included for procedure 1, 2 patients for optical imaging with CLE, and 2 with OCT. For procedure 2, 10 patients scheduled for RALP will be included, 5 of these patients will be imaged by CLE, 5 patients by OCT. Patients will be recruited in the AMC Hospital and VU Medical Center (Amsterdam, the Netherlands), and study procedures will be performed in both institutions. To increase the focal targeting of a PCa lesion, patients included in procedure 2 should have prostate mpMRI data available before the RALP with a visible (>5 mm) and suspect (PI-RADS v2: ≥3) region of interest. The other inclusion and exclusion criteria are listed in Textboxes 1 and 2, respectively. These sample sizes are based on prior publications and comply with the IDEAL 2a recommendation: low number of selected patients [29,30,32].

### Study Procedures

#### Procedure 1: Transperineal Template Mapping Biopsy (4 Patients, 2 Confocal Laser Endomicroscopy Imaging and 2 Optical Coherence Tomography Imaging)

**Figure 5:**
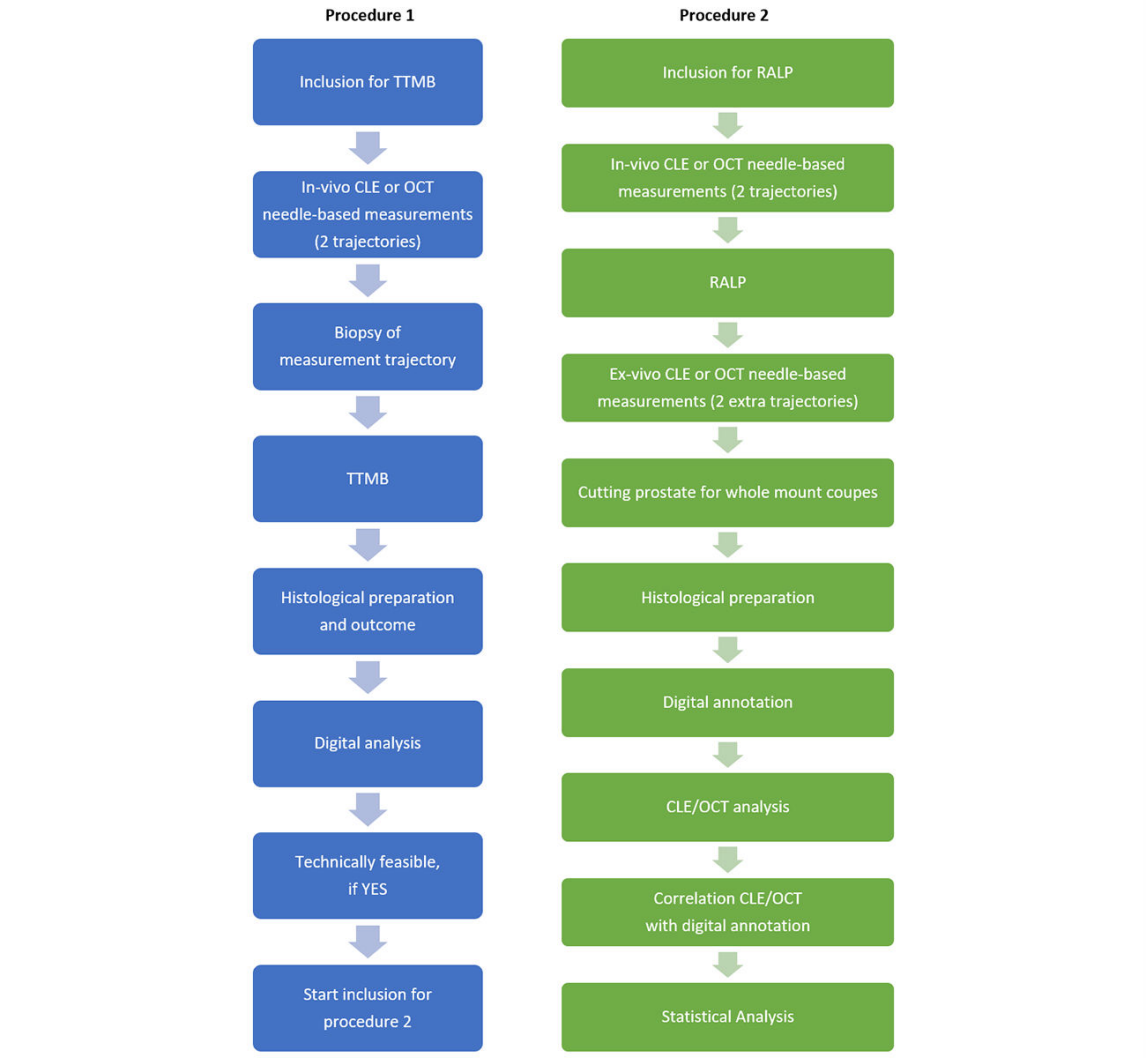
Flowchart procedure 1: transperineal template mapping biopsy (TTMB) study design; flowchart procedure 2: robot-assisted laparoscopic prostatectomy (RALP) study design. CLE: confocal laser endomicroscopy; OCT: optical coherence tomography.

The standard TTMB protocol is performed using local spinal or general anesthesia, and patients are positioned in the lithotomy position. Hereafter, the biopsy stepper is placed using a stabilizer and table mount. A clinical ultrasound scanner (HI VISION Preirus, Hitachi Medical Systems, Japan) with the biplanar probe (EUP-U533, Hitachi Medical Systems, Japan) and the endocavity balloon is used. After transrectal probe placement, dimensions and prostate volume are measured including checking of the pubic arch interference. The perineum is cleaned for surgery and draped. A sterile, disposable (brachy) template grid, consisting of rows and columns with holes spaced 5 mm apart, is used to guide the imaging probe/biopsy needle. The optical imaging acquisition is then started. As the CLE measurement technique differs from the OCT measurement technique; both techniques are described separately below. The measurement trajectories will be mapped with the ultrasound console. A corresponding biopsy will be taken following the same trajectory as the focal imaging technique (CLE or OCT). When the CLE or OCT measurements are performed, the standard biopsy cores will be taken, and the procedure is finished. Flowchart of procedure 1 is displayed in Figure 5.

**Inclusion criteria.**

To be eligible to participate in this study, a subject must meet all of the following criteria:

- Age ≥18 years
- Signed informed consent
- Multiparametric magnetic resonance imaging data are available (only for procedure 2)
- Visible (≥5 mm diameter) and suspect (prostate imaging reporting and data system, PI-RADS v2: ≥3) region of interest (only for procedure 2)

**Exclusion criteria.**

A potential subject who meets any of the following criteria will be excluded from participation in this study:

- Patients with a known allergic reaction to fluorescein
- Documented acute prostatitis or untreated urinary tract infections
- No ability to stop anticoagulant or antiplatelet therapy
- Medical history of a (potential) bleeding disorder
- Major concurrent debilitating illness or American Society of Anaesthesiologists Physical Status Classification System ≥4
- Chemotherapy for prostate cancer
- Androgen deprivation therapy within last 6 months
- Has any medical condition or other circumstances which would significantly decrease the chances of obtaining reliable data, achieving study objectives, or completing the study
- Is incapable of understanding the language in which the information for the patient is given

##### Confocal Laser Endomicroscopy Measurement Technique

For the CLE measurement, 0.5 mL of fluorescein (2.5% fluorescein diluted in saline) is intravenously injected for contrast. The CLE probe is inserted using a 17-gauge trocar needle. When the CLE is in contact with prostate tissue, the measurement begins; while recording, the probe and needle are pushed from apex to base. During this push and scan technique, the probe stays in contact with the tissue.

##### Optical Coherence Tomography Measurement Technique

The OCT probe is inserted through a 17-gauge trocar needle. The needle is placed at the end of the measurement trajectory. Then, the trocar needle is pulled back, so the probe is in contact with the surrounding tissue. When the probe is in contact, an OCT measurement will be made. The measurement is performed from base to apex.

#### Procedure 2: Robot-Assisted Laparoscopic Prostatectomy (10 Patients, 5 Confocal Laser Endomicroscopy Imaging and 5 Optical Coherence Tomography Imaging)

In the operating theater, before the RALP, the CLE or OCT measurements will be obtained in the same fashion as in procedure 1. Dimensions of the prostate will be measured on the ultrasound console. Following marked regions from the mpMRI, the CLE or OCT measurements will be made following the technique described earlier. After measurement, a plastic cannula will be left in the specific trajectory as a localization marker. This marker shows the measurement location necessary for analysis once the prostate has been removed. After the cannula placement, the TRUS-probe and stepper will be removed, and the standard RALP can start. The plastic cannulas will remain in place during the removal of the prostate. Figure 5 shows the flowchart of procedure 2.

#### Multiparametric Magnetic Resonance Imaging

MpMRI is a combination of T2-weighted MR imaging, diffusion-weighted MR imaging, and dynamic contrast-enhanced MR imaging. MpMRI of the prostate enables detection of the prostate tumor with reasonable sensitivity and specificity values [33]. MpMRI will be evaluated by a uroradiologist for evidence of PCa localization according to the PI-RADSv2 criteria [34].

### Data Analysis

Demographic and disease-specific characteristics of the study populations (eg, age, PSA, DRE, biopsy localization, tumor location on imaging and pathology, tumor size, and Gleason score) will be collected. First, CLE and OCT data will be evaluated in a qualitative way. The data will be compared with histopathology, and characteristics of the following different tissues in the prostate will be described; benign glands, cystoid atrophy, regular atrophy, stroma, malignant tissue using the Gleason score, inflammation, and fat. The data will be obtained and analyzed by nonblinded investigators, and subsequently, investigators blinded to the results will interpret all individual measurements for diagnostic evaluation. An independent uropathologist, blinded for the CLE and OCT results will perform the histopathology. Second, OCT data will be analyzed quantitatively. We will determine and report the attenuation coefficient, the decay of light in tissue, per tissue type in the prostate [15,35].

### Safety

The investigators will monitor patient safety. They can withdraw a patient from the study for medical reasons. In accordance to section 10, subsection 4, of the “Wet Medisch-Wetenschappelijk Onderzoek met Mensen” (medical research involving human subjects act in the Netherlands), the investigators will suspend the study if there is sufficient ground that the continuation of the study will jeopardize patient’s health or safety. The investigators will notify the accredited IRB if this is the case. In case of an adverse event or serious adverse event, the responsible authorities will be informed.

### Benefits and Risks

As the patients included in this study are already scheduled for radical prostatectomy or TTMB, no direct benefit exists. The results of this study may be relevant for patients in the future for PCa diagnosis, grading, and staging. CLE and OCT are promising imaging techniques that in conjunction with the TRUS/mpMRI fusion guided biopsy procedure can provide real-time, high-resolution 3D microscopic imaging and tissue characteristics of PCa.

Previous in vivo studies using CLE or OCT did not report any adverse events, and these modalities are performed by needle guidance with the same diameter or smaller as the standard biopsy needles. In case of a RALP, 2 plastic cannulas will be placed using an intravenous needle. The plastic cannulas will stay in the prostate during the surgery and could, therefore, harbor an increased risk for infection, positive surgical margin rate, or other (unknown) complication during surgery. Standard antibiotic prophylaxis (ciprofloxacin) will be administered 2 h before surgery to reduce the risk of infection. The proposed needle-based imaging techniques also imply a puncture into the prostate and, therefore, have a risk of complications such as bleeding. However, bleeding is believed to be limited as only 2 needles will be placed; complications will be documented and critically analyzed in this safety and feasibility study. Fluorescein is a commonly used fluorescent dye that will be administered intravenously through an intravenous cannula. Previous reports have proven that it is safe and easy to administer [36-38]. Possible side effects include nausea, vomiting, abnormal taste sensations, thrombocytopenia, and allergic reactions. Patients with a known allergic reaction to fluorescein are excluded from participation in this study. Standard care and pathological evaluation as stated by the internal protocols will not be affected in this study. In conclusion, we believe that the burden and risk associated with participation in this study are limited.

## Results

Presently, recruitment of patients is ongoing in the study. Results and outcomes are expected in 2019. Summarized raw data will be made available through publication in an international peer-reviewed medical journal.

## Discussion

This protocol describes the first in vivo study for needle-based optical biopsies using CLE and OCT in the prostate. Both techniques may enable real-time pathological information by showing cellular characteristics on CLE images and microarchitecture on OCT images. The study comprises 2 parts: feasibility of the technology and comparison with histology.

This first part contains multiple similarities with the protocol of Wagstaff et al using needle-based OCT in the kidney [16]. Using ultrasound guidance, a trocar needle was placed to guide the OCT needle and subsequently the standard biopsy needle, both sampling the same location. Instead of using a trocar needle, in this protocol, a transperineal grid will be used as a guidance tool. This transperineal grid will allow targeting of the suspected lesion based on cognitive fusion with prostate mpMRI, which has shown to be as good as automatic fusion [39]. The expected burden for the patients is thought to be minimal by using only 2 extra needles; by target placement of the 2 needles, the possibility of sampling the lesion is as high as possible.

The second part of the protocol enables one-to-one comparison of in vivo data with histology for both CLE and OCT. Our approach is similar to the approach of Muller et al [28] that compared ex vivo needle-based OCT measurements of radical prostatectomy specimens with histology by cutting through the measurement trajectories. In our measurements, data will be obtained from in vivo tissue, in which red blood cells will absorb and scatter light different from regular cells. Due to the perfusion of prostate tissue, the acquired data will most probably differ from the ex vivo measurements [29,40]. Nonetheless, this study will enable us to understand the in vivo OCT and CLE images and challenges in co-localization of acquired in vivo data with ex vivo histology.

In the described study, safety and feasibility of both imaging techniques are assessed in patients under operating theater circumstances with general or spinal anesthesia. Although safety and feasibility could be different in patients under local anesthesia in an outpatient setting, we expect that both proposed needle-based imaging techniques are easily translated to an outpatient setting as both use equal or smaller diameters as biopsy guns and both are designed to be integrated into the outpatient workflow.

Several studies have provided in vivo images of CLE, but do not show a comparison with histology or an in-depth interpretation of the prostate images [38]. On the basis of histopathology, it is expected that benign prostate tissue differs in extracellular structure compared with malignant prostate tissue. The fluorescein, provided by intravenous injection, gives contrast to the extracellular matrix on the CLE images, which could potentially allow to discriminate between benign and malignant prostate tissue. The described protocol will compare histology and CLE to provide knowledge of visual characteristics on CLE images.

Locating and recording the position of the in vivo measurements is difficult and will be less precise than an ex vivo measuring environment. The in vivo measurement locations will be mapped by ultrasound, and the measurement trajectory will be marked for ex vivo histology comparison. Regardless of this precise measurement mapping, the size and shape of the prostate will change after removal and formaldehyde fixation and could cause potential correlation errors [41]. These changes in dimensions will be recorded by measuring the size of the in vivo prostate by ultrasound and when fixated, to be able to correct for prostate shrinkage. During the comparison of in vivo measurements and ex vivo histopathology, the measurement trajectory will be scaled. The length of the measurement trajectory will be scaled following the shrinkage of the prostate. Shrinkage of tissue over the trajectory is not uniform, but this is in our opinion is the best available option to correct for the shrinkage.

This study is an essential first step for the clinical evaluation of optical imaging in PCa diagnosis. In the clinic, a tool for optical histology could potentially guide a biopsy needle with instant feedback of the region of interest for reliable diagnosis and treatment of PCa.

## Abbreviations

CLE: confocal laser endomicroscopy
IRB: institutional review board
mpMRI: multiparametric magnetic resonance imaging
OCT: optical coherence tomography
PCa: prostate cancer
PI-RADS: prostate imaging reporting and data system
PSA: prostate-specific antigen
RALP: robot-assisted laparoscopic prostatectomy
TRUS: transrectal ultrasound
TTMB: transperineal template mapping biopsy
